# Kinematic Analysis During Straight Line Free Swimming in Horses: Part 1 - Forelimbs

**DOI:** 10.3389/fvets.2021.752375

**Published:** 2021-10-14

**Authors:** Emma Santosuosso, Renaud Leguillette, Tatiana Vinardell, Silvio Filho, Shannon Massie, Persephone McCrae, Sarah Johnson, Campbell Rolian, Florent David

**Affiliations:** ^1^Faculty of Veterinary Medicine, University of Calgary, Calgary, AB, Canada; ^2^Equine Veterinary Medical Center, Member of Qatar Foundation, Doha, Qatar; ^3^College of Health & Life Sciences, Hamad Bin Khalifa University, Member of Qatar Foundation, Doha, Qatar; ^4^Al Shaqab - Endurance Department, Member of Qatar Foundation, Doha, Qatar

**Keywords:** swimming, kinematics, horses, front limb, rehabilitation, range of motion, joints, mobility

## Abstract

**Background:** Swimming is used for rehabilitation and conditioning purposes in equine sports medicine despite the lack of understanding of equine swimming kinematics. The aim of this study was to assess forelimb joints kinematics (elbow, carpus, and fetlock) in swimming horses. The specific objectives were 1- to calculate and compare joint angles in swimming vs. passive mobilizations (PM), 2- to determine joint angular velocities during a swimming stride cycle.

**Methods:** Eleven elite endurance horses swam in a 100-m straight pool. Underwater (swimming) and overground (PM) videos were recorded from the horses' left side. Joint markers were applied on the lateral hoof wall, lateral metacarpal epicondyle, ulnar carpal bone, lateral humeral epicondyle, and the greater tubercle of humerus, from which elbow, carpus and fetlock angles, and angular velocities were obtained. As a reference, maximal fetlock, carpus, and elbow flexion/extension angles were determined during PM overground. Differences between angle extrema, angular velocities and range of motion (ROM) were compared.

**Results:** Carpus and fetlock ROM were significantly smaller (*p* < 0.001) during swimming when compared with PM, while there was no difference in elbow ROM between both situations. The carpus had the greatest ROM of all joints during swimming. Absolute angular velocities values of all joints during swimming were greater during retraction than protraction (*p* < 0.001). When compared to other joints during protraction, the carpus joint reached the highest angular velocity.

**Conclusion:** Swimming, as a rehabilitation exercise, has the potential to benefit horses where great elbow ROM with a moderate carpus and fetlock extension are wanted.

## Introduction

The potential therapeutic benefits of hydrotherapy have been studied previously in humans, as buoyancy and viscosity enable increased muscle activation and reduced joint loading (1–5). Similarly, aquatic exercise programs with water treadmills and swimming pools are used in horses for rehabilitation and conditioning (6–9). However, evidence supporting the benefits of equine swimming as a form of cross-training and rehabilitation is scarce in the literature (8, 9). Instead, research efforts have focused on the physiological and kinematic effects of equine water treadmill exercise (10–12). Changes in gait have been documented during water treadmill exercise in deep water with an increase in the range of motion (ROM) of the carpus, tarsus and fetlock joints (12, 13). It has also been shown that water treadmill exercise causes a decrease in angular velocity of the forelimb when compared to dry conditions, in both the protraction and retraction phases - likely due to increasing drag created by water (14).

Although the physiological response (heart rate, cardiac arrhythmias, respiratory rate, blood pressure) of swimming horses have previously been well-documented (15–21) studies assessing musculoskeletal function during swimming in horses are rare, likely because of the challenges associated with underwater data collection. Little is known about horses' swimming movements, though it has previously been noted that there was great variability in swimming pattern between individuals (22). An electromyography study in horses has shown that swimming activity results in altered muscle activation compared to overground conditions (23). One of the potential drawbacks associated with swimming exercise in horses is the significant extension of the spine and an increased risk of injury due to exaggerated motion of legs (4). Circular pools were also suggested to have a potential detrimental effect on the limbs' kinematics during repeated swimming in circle (22).

A study comparing the ROM of hindlimb joints during swimming has been conducted in dogs following surgery for cranial cruciate ligament repair and healthy controls (24). In both groups, ROM of the stifle and tarsus were greater during swimming than during walking on a dry treadmill. Similar ROM data are not available in horses. However, these findings suggest that swimming exercise could provide benefits for the rehabilitation of horses with limb injuries when reduced axial loading, coupled with increased ROM of certain joints, is preferred to low-speed overground exercise.

The present study aims to describe the kinematics of the forelimb (elbow, carpus and fetlock joints) during free (untethered) swimming in a straight pool. A companion manuscript describes the kinematics of the hindlimb (25). The specific objectives of the present study were: (1) to compare forelimb joint angles during swimming with those obtained during passive flexion and extension, (2) to determine angular velocity during a swimming cycle, for each of the examined joints, and (3) to compare the angular velocity for each joint during the protraction and retraction phases of the swimming cycle. We hypothesize that joints' ROM would be greater during passive flexion and extension compared to swimming, that the angular velocity would differ between joints (with greater values obtained for more distal joints), and that absolute values of angular velocity would be greater during retraction than protraction.

## Materials and Methods

The study was approved by the Institutional Animal Care and Use Committee of the Equine Veterinary Medical Center, a member of Qatar Foundation (EVMC-2020-1135) and performed at the Al Shaqab Equine Exercise Center where the straight pool is located.

### Horses

A group of 11 healthy, elite endurance horses (7 geldings and 4 mares; mean age ± Standard Deviation (SD) 13.8 ± 3.2 years; weight 427 ± 41.1 kg) were enrolled in the study. Each horse was confirmed to be free of lameness based on history and a detailed clinical examination. The horses used in the study were familiar with the pool facilities and had all previously undergone at least 2 months of training that included regular swimming sessions.

### Experimental Protocol

Data were collected in an indoor, 100 m long and 3 m deep, straight pool that allowed free swimming in a straight line over a 70 m distance. Horses were only restrained by a halter and loose lead rope by a handler from the right side of the pool. The pool water was transparent and lit from both above and underwater. Swimming speed was recorded using a timer and distance markers along the side of the pool. Horses were allowed to swim at their preferred speed without interference from the handler. Videos were recorded from the left side, with cameras placed at least 25 m away from the free swimming start zone to ensure that horses had reached their preferred swimming speed. The two-dimensional (2D) movements were recorded using two underwater digital video cameras^1^ with an acquisition rate of 60 frames/sec, a resolution of 1,440 pixels. The cameras were positioned horizontally (confirmed with a level) by a diver on the left wall of the pool using suction cups at 50 cm under the water surface. The two cameras were set at 2 m distance from each other. Before each recording, a calibration ruler was put in front of each camera's field of view, at approximately the same distance as the left forelimb of the horse would be during swimming. The handlers were instructed to gently guide the horses so that they would swim closer to the far wall of the pool (relative to the cameras) to ensure proper framing of the entire horses' legs.

**Figure 1:**
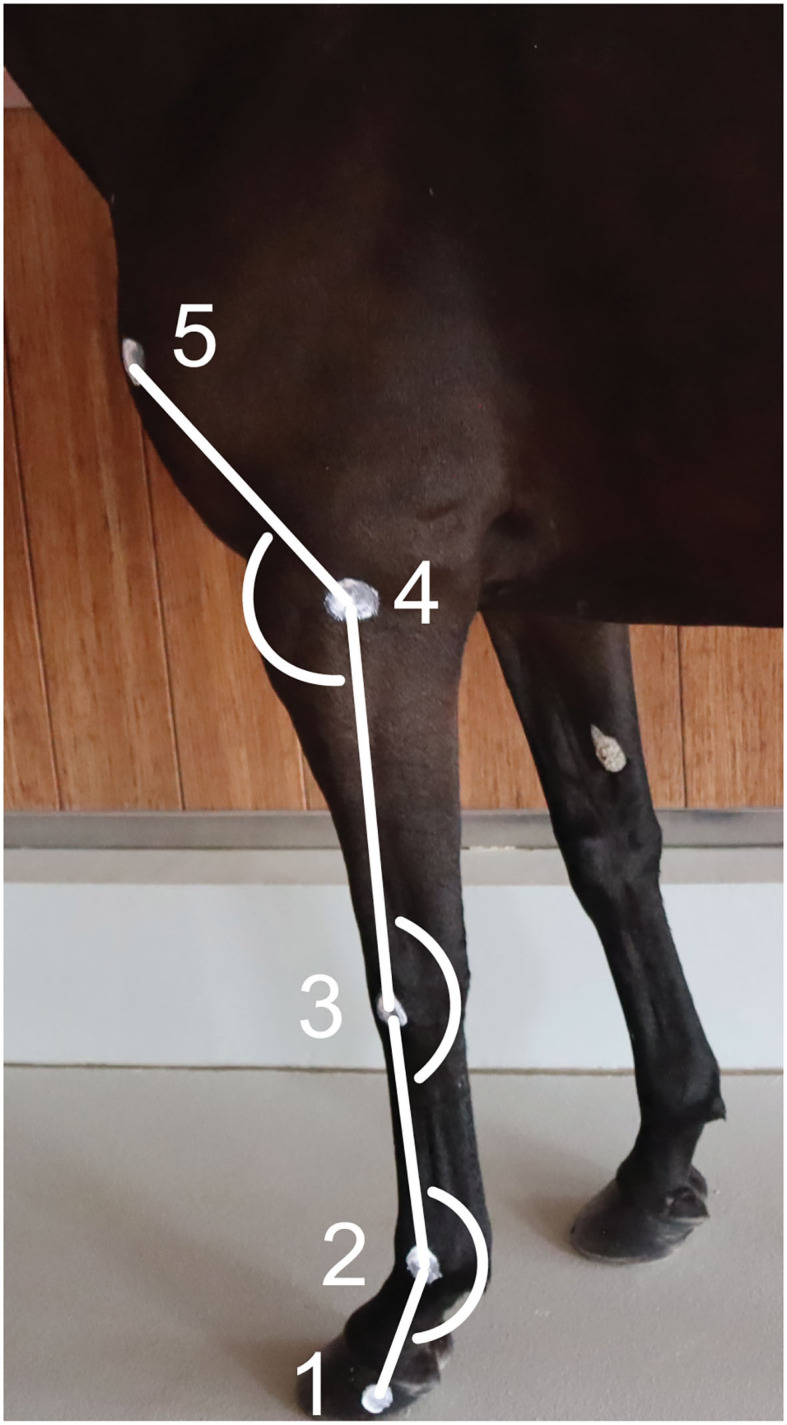
Position of the markers on the forelimb's anatomical landmarks and angles calculated. (1) Lateral hoof wall, (2) Lateral metacarpal epicondyle, (3) Ulnar carpal bone, (4) Lateral humeral epicondyle, and (5) Greater tubercle of the humerus.

Two-centimeter diameter zinc oxide cream^2^ round markers (12, 13) were applied on the left forelimb at the following level of the (1) lateral hoof wall (distal interphalangeal joint), (2) lateral metacarpal epicondyle, (3) ulnar carpal bone, (4) lateral humeral epicondyle, and (5) greater tubercle of humerus (Figure 1) (26). The fetlock, carpal and elbow joint angles were defined, respectively, as the palmar angle between segments joining markers 1, 2, and 3; the palmar angle between segments joining markers 2, 3, and 4; and the dorsal angle between segments joining markers 3, 4, and 5, respectively (Figure 1). The ROM was calculated as the difference between the maximum flexion and extension for each joint.

**Figure 2:**
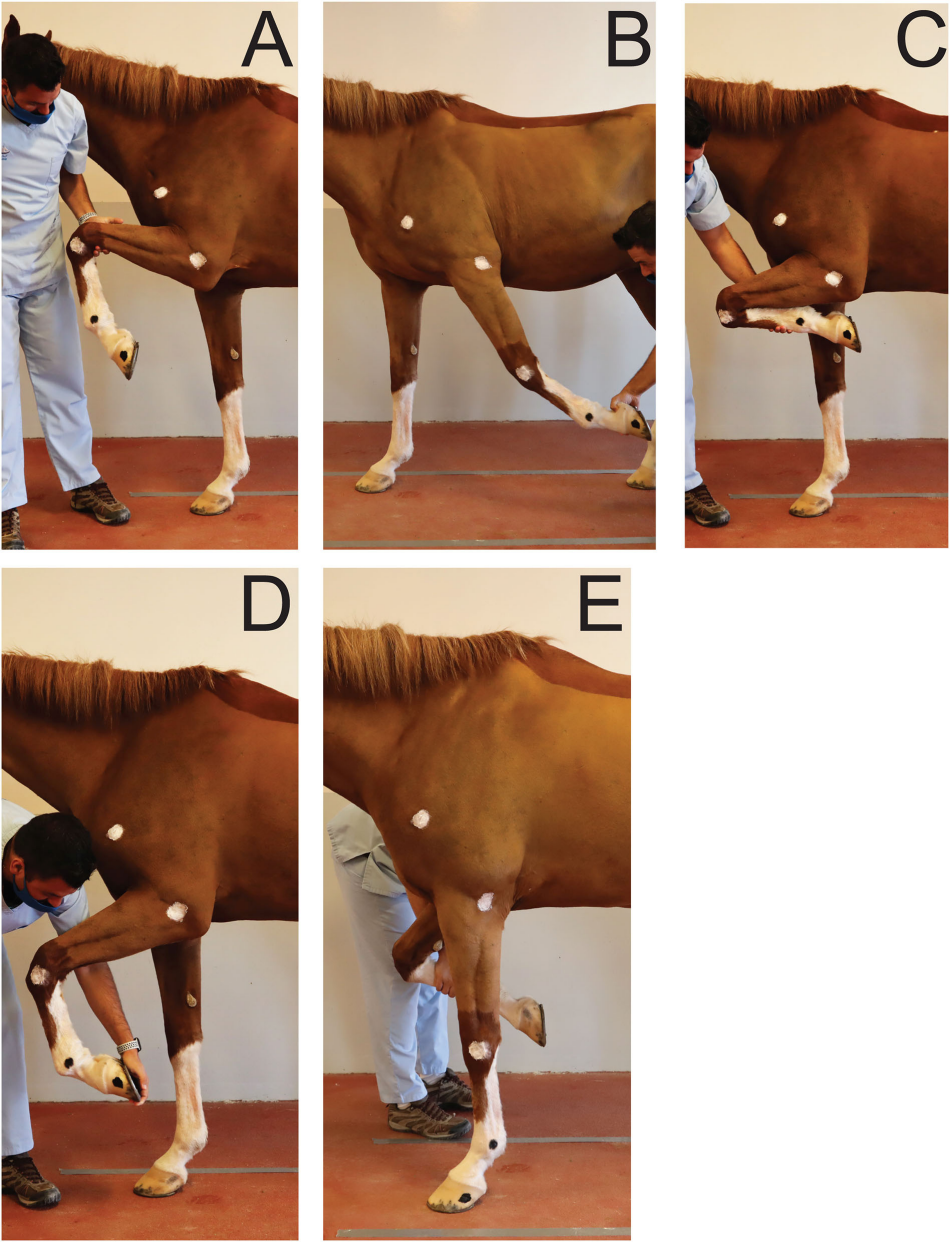
Positions obtained for maximal flexion and extension of the joints of interest during passive mobilization. **(A)** Maximal elbow flexion; **(B)** Maximal elbow extension; **(C)** Maximal carpus flexion; **(D)** Maximal fetlock flexion; **(E)** Maximal carpus and fetlock extension.

To establish a reference, for each joint, maximal passive flexion and extension were recorded for each horse in a static position overground (Figure 2). Two operators were trained to ensure standardization of maximal passive flexion and extension of the joints outlined above. Horses were standing over ground markers in a delimited area. A video camera^3^ on a tripod was used to record the videos at 2.40 m from the horse's left side and at 1.18 m from the ground. For the extension of the (left) fetlock and carpus, the joint angles were recorded while the horse was bearing full weight on the left forelimb in a vertical position (Figure 2E). For the extension of the elbow, the forelimb was elevated and the radius retracted caudally until maximal extension was achieved (Figure 2B). For the flexion of the joints, the forelimb was elevated, and the handler proceeded to flex or lift until maximal flexion of the joints were achieved (Figures 2A,C,D). The entire procedure was repeated three times and by each operator in order to average intra- and inter-operators variability. The order in which the joints were assessed was randomized.

### Kinematic Analysis of Swimming

**Figure 3:**
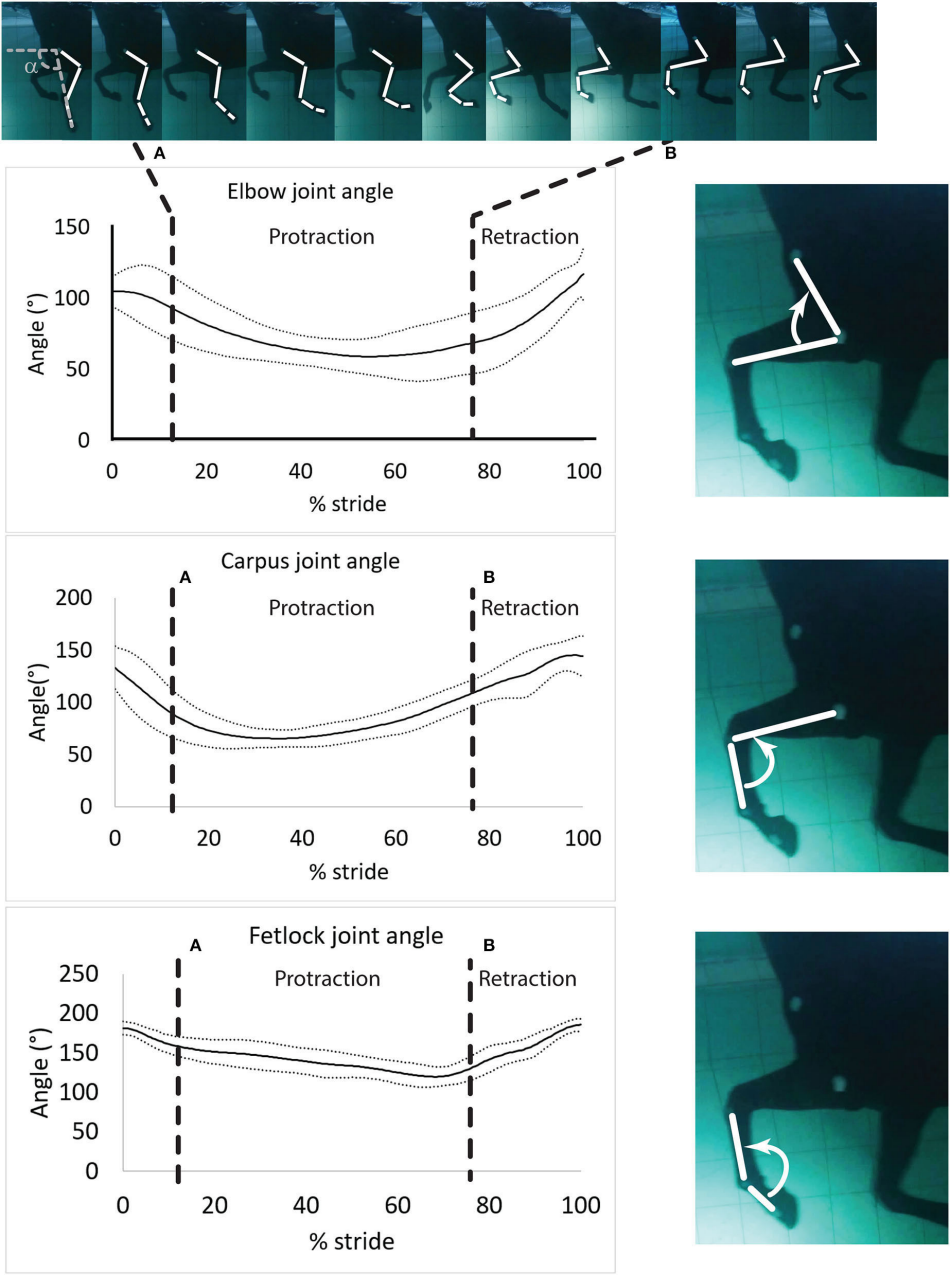
Mean ± SD of the elbow, carpus and fetlock joint angles during a typical and complete swimming stride cycle (protraction and retraction). **(A)** Start of protraction phase; **(B)** Start of retraction phase. α: protraction-retraction angle. The arrow indicates the flexion of the joint of interest on the side illustration.

Videos from each camera were analyzed independently (i.e., not as a continuation of the swimming strides). One stride cycle was defined as the period between two consecutive instances of maximum fetlock extension. Therefore, the onset of the stride cycle was defined as the instant when the fetlock angle was maximal (Figure 3).

Swimming speed was calculated from the distance the horse covered during the duration of time, as determined by the frame rate. The videos were reviewed for quality and only those with clear visibility of all skin markers were used for analysis. Manual marker tracking was carried out by the same operator using a 2D motion analysis software^4^ as previously described (27, 28). The 2D coordinates obtained in the X- and Y-axes were exported to a spreadsheet. Manual interpolation was performed to normalize data and to express time as a percentage of total stride duration. Angular velocity was calculated using first derivatives of the angular displacement (Equation 1).


$$\begin{array}{l} \omega =\frac{\text{Δ}\theta }{\text{Δ}t} \end{array}$$

*Equation 1: Angular velocity equation*

ω*: angular velocity;* Δθ*: angle difference;* Δ*t: time difference*.

Angular velocities were assigned positive values when the angle difference or the angular velocity difference was positive (extension of the joint) and negative otherwise (flexion of the joint) (Figure 3).

Angular velocity data for the joint angles were exported into a computing software^5^ and a local regression (LOESS function) was applied with a constant span (29). Maxima, minima and joint ROM as well as angular velocities maxima (maximum positive angular velocity during extension) and minima (maximal negative angular velocity during flexion) were normalized with respect to time of a stride cycle to enable comparisons among horses. The starting point (T0) of the stride cycle was defined when horses reached maximal fetlock extension. Mean angular velocities for the joint angles were also calculated during the limb protraction and retraction phases of the swimming stride cycle.

### Kinematic Analysis of Passive Mobilization

For each horse, six brief (3–4 s long) videos were imported into the 2D motion analysis software KINOVEA and the mean of the maximal and minimal joint angles obtained from the two operators were calculated using the same methods described above. The overall results were expressed as ratios of the maximal flexion, extension and ROM obtained during swimming relative to those obtained during passive mobilization. Ratios greater than one indicated that flexion, extension or ROM was greater during swimming vs. passive mobilization.

### Statistical Analysis

A Shapiro-Wilk test was performed to assess normality of kinematic parameters. A one-way repeated measure analysis of variance (ANOVA) followed by a Tukey *post-hoc* test was performed to compare the differences between minimal and maximal angles, ROMs and minimal and maximal (peak) angular velocities of each joint. Values obtained during passive mobilization and swimming were compared using a one-way ANOVA. Mean angular velocities obtained during protraction were compared to values obtained during retraction using a paired *t*-test. Statistical significance was set at *p* < 0.05 and all results were presented as mean ± SD, as datasets were normally distributed. Inter-observer agreement was assessed using a Bland-Altman test (limit of agreement: 95% confidence limit).

## Results

### Swimming Cycle Parameters

The data from one horse were not included in the study due to an atypical swimming style. This mare relied solely on her hind quarters to swim and kept her front limbs in a semi-flexed position with very rare swimming movements (see Supplementary Figure 1A). Between two and four complete stride cycles were recorded for each of the 10 remaining horses, for a total of 30 strides. Seven of the total strides recorded were not analyzed due to the inability to consistently visualize all markers. A total of 23 complete stride cycles were used for analysis (2 horses with 1 stride cycle analyzed, 4 horses with 2, 3 horses with 3 and 1 horse with 4). All swimming stride parameters are presented in Table 1.

**Table 1:** Forelimb stride cycle parameters in horses during untethered swimming (n = 10).

**Parameters**	**Mean ± SD**
Speed (m/s)	1.1 ± 0.2
Cycle duration (s)	1.1 ± 0.2
Protraction phase (s)	0.8 ± 0.2
Retraction phase (s)	0.4 ± 0.08
Swimming stroke length (m)	1.2 ± 0.2

### Joint Angles

Angle-time diagrams (as percentage of the swimming stride cycle) obtained for the fetlock, carpus, and elbow joints are provided in Figure 3. The protraction phase was from 13.5 to 77.5% of the stride (Figure 3). During the protraction phase of the forelimb, the greatest peak in flexion was first reached by the carpus (early protraction), then the elbow (mid protraction) and finally the fetlock (end protraction) (Table 2 and Figure 3). The maximal flexion and extension of the joints were significantly different from each other (Table 2) and the carpus swimming ROM was significantly greater than the swimming ROM of the fetlock (*p* = 0.002) and elbow (*p* = 0.002).

**Table 2:** Joint angle parameters in horses during swimming (*n* = 10).

	**Elbow**	**Carpus**	**Fetlock**
**Angles**			
Maximal extension (°)	125.9 ± 15.7^a,b^	156.7 ± 12.6^a,c^	188.3 ± 5.3^b,c^
% stride at maximal extension	3.3 ± 3.1	88.4 ± 29.4	82.6 ± 38.2
Maximal flexion (°)	52.8 ± 7.6^d,e^	60.9 ± 7.0^d,f^	111.4 ± 9.1^e,f^
% stride at maximal flexion	55.8 ± 12.9	20.5 ± 8.7	69.9 ± 9.7
Angular ROM (°)	73.1 ± 16.5^g^	98.5 ± 16.6^g,i^	78.3 ± 14.8^i^
**Angular velocity**			
Maximal (positive) angular velocity in retraction (°/s)	328.8 ± 167.1	520.0 ± 171.1	477.3 ± 131.8
% stride at maximal (positive) angular velocity	83.5 ± 29.9	74.3 ± 20.4	83.9 ± 8.2
Maximal (negative) angular velocity in protraction (°/s)	−300.0 ± 100^j^	−560.0 ± 210^j,k^	−320.1 ± 180^k^
% stride at maximal (negative) angular velocity	22.3 ± 17.5	18.1 ± 29.1	26.1 ± 33.1
Mean (positive) angular velocity in retraction (°/s)	173.1 ± 82.8	131.4 ± 57.6 ^n^	226.3 ± 49.4 ^n^
Mean (negative) angular velocity in protraction (°/s)	−53.7 ± 21.0^l^	29.6 ± 17.8^l,m^	−55.3 ± 18.3^m^

*Similar letters indicate significantly different values between two joints (p < 0.05). Protraction phase is from 13.5 to 73% of the stride. Angles measured are presented in Figure 1*.

### Angular Velocity During Protraction and Retraction Phases

The absolute value of the angular velocities was significantly greater during retraction than during protraction (*p* = 0.003) for all joints (Table 2).

Maximal negative angular velocity was reached in the early phase of protraction (Table 2), indicating that the flexion velocity of these joints was the fastest at this stage of the stride. For both phases, peak (extrema) angular velocities were first reached by the carpus, followed by the elbow and the fetlock (Table 2).

During retraction, the fetlock had the greatest (positive) angular velocity. During protraction, the elbow and the fetlock had a significantly greater (negative) angular velocity compared to the carpus (Table 2).

### Comparison Passive Mobilization-Swimming

Nine out of 10 horses with swimming strides analyzed were available for the assessment of passive mobilization as one was lost to follow up. The Bland-Altman scatter plots revealed a good agreement between handlers during passive mobilization. The ROM of the carpus and fetlock joints were greater (*p* < 0.001 for both) during passive mobilization (+59.9% and +35.6% respectively) than during swimming (Table 3). The carpus was the only joint where the maximal flexion was significantly (*p* < 0.001) smaller during swimming (−50.5%) than during passive mobilization (Table 3). The carpus and fetlock showed significant (*p* = 0.0005 and *p* < 0.0001 respectively) smaller maximal extension during swimming (−14.4% and −12.8% respectively) than during passive mobilization (Table 3).

**Table 3:** Comparison between ROM obtained during passive mobilization and swimming (n = 9).

	**Elbow**	**Carpus**	**Fetlock**
**Passive mobilization**			
Maximal flexion	58.1 ± 8.2^d,e^	30.6 ± 4.9^d,f^	120.4 ± 7.7^e,f^
Maximal extension	129.8± 9.4^a,b^	184.1 ± 3.1^a,c^	218.2 ± 5.1^b,c^
ROM	71.7 ± 11.6^g^	153.5 ± 4.3^g,i^	97.7 ± 5.9^i^
**Ratios swimming/passive mobilization**			
Ratio flexion	0.9 ± 0.3	1.9 ± 0.1^*^	1.0 ± 0.1
Ratio extension	1.0 ± 0.1	0.8 ± 0.1^*^	0.8 ± 0.0^*^
Ratio ROM	0.9 ± 0.2	0.8 ± 0.2^*^	0.9 ± 0.2^*^

*Ratio flexion (Extension, ROM, respectively), indicates the ratio of maximal flexion (Extension, ROM) during swimming to maximal flexion (Extension, ROM) obtained during passive mobilization*.

* *indicates a statistically significant difference (p < 0.05) between numerator and denominator of the ratio. Similar letters indicate significantly different values between two joints (p < 0.05)*.

## Discussion

This study is the first to describe the underwater kinematics of the equine forelimb during swimming. Carpal and fetlock swimming ROM were reduced compared to passive mobilization due to reduced joint extension, likely because of the absence of a weight-bearing stance phase during swimming.

In the present study, two horses displayed significant variations in the typical swimming style observed in the majority of horses. As shown in the Supplementary Figure 1A, one horse did not display any significant forelimb motion and was excluded from analysis. Another horse maintained a complete leg extension during part of the protraction phase (Supplementary Figure 1B). The impact of these atypical swimming style variations on swimming efficiency remains to be determined.

Swimming speed in the present study (1.1 ± 0.2 m/s) was similar to speeds previously measured during untethered swimming in Thoroughbred horses (1.06 m/s) in an oval shaped pool (30). Comparisons between swimming speed and water treadmill exercise speeds are not relevant as speed was imposed in all water treadmill studies (14, 31, 32). It remains to be determined if the swimming speed varies with the number of laps performed during a swimming session (effect of initial stress or fatigue), with the horse's experience with this aquatic exercise (progressive improvement in swimming efficiency) or with external stimuli (effect of handler's encouragement or of another horse swimming in a parallel corridor).

### Joint Angles

Maximal flexion, extension and ROM were compared between swimming and passive mobilization. Rehabilitation protocols typically utilize passive flexion early on, often followed by walking in-hand combined with low impact exercise such as swimming or underwater treadmill exercise (33). Intuitively, it would be expected that joint angles during swimming would be smaller than passive flexion-extension angles, as the passive mobilization is forced, reaching maximum amplitude for a given joint. Although this was true for the carpus and the fetlock, this was not the case for the elbow, where ratios close to 1 were obtained, indicating a similar ROM for the elbow between swimming and passive mobilization. It is possible that passive extension of the elbow was underestimated as horses do not always cooperate when passive radius retraction is performed, despite the absence of elbow pain. Extension of the fetlock and carpus, and flexion of the carpus were clearly greater during passive mobilization while fetlock flexion was equivalent between the two rehabilitation activities. During walking, Galisteo et al. (34) showed that maximal extension of the fetlock and carpus was achieved during the retraction portion of the stance phase. Therefore, in the absence of ground contact during swimming, it was expected that the extension of the carpus and fetlock during swimming would be lower than during walking overground (34). For horses stagnating during the rehabilitation of an injury and showing pain or avoidance/defensive behavior during passive flexion/extension of a given joint, the introduction of swimming exercise may help to regain some ROM in a safe way.

We found that the maximal flexion of all joints occurred during the protraction phase and is first achieved by the carpus, followed by the elbow and then the fetlock. This is similar to what has previously been described during trotting overground (35). The maximum extension of the fetlock, carpus and elbow were achieved at the end of the retraction phase, similar to the sequence observed at a trot (35). However, when trotting overground, the maximal extension of the fetlock is first reached during the retraction phase while the hoof is vertical (right before heel on) (35), which was not observed during swimming. Although the sequence of flexion and extension of the three joints within the forelimb presents similarities with ground exercise (36), there were key differences in joint angles during swimming. The maximal extension of the three joints in the present study were lower than those described overground, regardless of the gait (37). The maximal extension of the carpus and the fetlock were also lower than those published during water treadmill exercise, regardless of water height (13). This is likely due to the absence of the stance phase and associated weight-bearing during swimming, compared to overground or water treadmill exercise. The buoyancy of water and the lack of ground reaction forces prevent the effect of weight-bearing on the fetlock and carpus, specifically limiting the typical passive extension of these joints (34). However, we found that the extension of the elbow and carpus was greater during swimming than what has been reported previously for water treadmill exercise (13, 32). The greater flexion observed in aquatic locomotion (either swimming or water treadmill exercise) is associated with an elevation of the forelimb (32). Increased flexion was hypothesized to increase the efficiency of motion in water by decreasing the surface in contact with the water and the hydrodynamic resistance during protraction (see Equation 2) (38). Other parameters than the size/shape of the leg such as the properties of the fluid, and the speed of the object can also affect the drag force.


$$\begin{array}{l} \;{\text{F}}_{\text{D}}=\frac{1}{2}\;C\;\rho \text{ A }\nu^{2} \end{array}$$

*Equation 2: Drag force equation*

*FD: Drag force; C: Drag coefficient; ρ: Water density; A: Cross sectional area; ν: Velocity of the object relative to the fluid.*

The increased flexion is almost certainly an active process – a previous study reported that the *flexor digitorum profundus* (FDP), which flexes the carpal and digital joints, had the most intense activity during swimming in horses (23).

Elbow and carpus ROM were greater during swimming than during overground walking or trotting (39, 40) and water treadmill exercise (13, 32), while fetlock ROM was decreased during swimming compared to what has been reported overground and in the water treadmill (32, 39, 40). Chronic dysfunction or pain in a region of the limb often results in ROM loss of one or several joints (41). Using swimming exercise to increase or restore normal ROM of a given joint is particularly interesting for rehabilitation purposes, as shown in dogs after cranial cruciate ligament repair (24). Specifically, the degree of carpal flexion (which contributes to the ROM) was greater in horses during swimming than during water treadmill exercise at any water height (5, 32). This can be exploited in the rehabilitation of wounds involving the dorsal carpus where loss of carpal flexion and associated post-injury stumbling are common complications of coronation injuries (42).

The decreased ROM of the fetlock during swimming was mainly due to a low degree of extension during swimming in the absence of a stance phase. This finding suggests that swimming could be utilized for early rehabilitation of orthopedic injuries involving the suspensory apparatus of the fetlock or the superficial digital flexor tendon, since they are put under high strain during extension in the stance phase. While some rehabilitation protocols may attempt to increase the ROM of certain joint, a reduction of fetlock extension may also be desirable, as shown with the use of a specific rehabilitation device (43). Therefore, other pathological conditions that could benefit from swimming protocols during the early post-injury phase include suspensory or distal sesamoidean ligament desmitis, flexor tendinopathies, sesamoid bone fractures, and fetlock joint overextension injuries. In humans the benefits of aquatic therapy have been demonstrated for orthopedic conditions such as osteoarthritis (44) and ligament injury (45). As shown in immobilized horses, bone quality and density loss can be expected (46). In an experiment conducted in osteoporotic rats, swimming activity was able to prevent the deleterious effects of inactivity on bone quality and even to further ameliorate it (significant increase of 43% in bone mass and 29% in bone strength) vs. immobilized controls (47). The beneficial effects of swimming on bone metabolism via the expression of the Wolff's law should therefore not be underestimated when long period of rest are required and warrant attention in horses to fill this knowledge gap.

### Angular Velocity

Significant differences between the angular velocities of the joints were observed, which indicates a difference in the importance of each joint in the swimming cycle and swimming efficiency in the horse.

The absolute value of the carpus angular velocity was the highest observed during protraction. Since angular velocity is defined as the angular displacement (°/s) over time, our data suggest that the carpus joint, by undergoing rapid flexion, would contribute the most in reducing the water resistance enhancing the protraction of the forelimb. During retraction, mean angular velocity of the fetlock was the highest of all the three joints, significantly higher than for the carpus.

There is currently no evidence of any relationship between the increased joint ROM (24) or increased angular velocities during swimming, compared to overground gaits, and their efficiency to enhance the height of the individual (48) during immersion in water. As angular velocities are an integral part of the calculations of joint power (moment x angular velocity), it is reasonable to assume that the greater angular velocity of these joints would generate increased power. This hypothesis and the observation reported in the present study are confirmed by great absolute value of joint moments observed during the second part of swing phase at trot (48). If no statistical analysis was performed to compare the difference of joint moment between the different phases of the swing phases, this compares to the beginning of the swing phase. Further data about limb segments weight and drag coefficient in the water, among other factors, would be necessary to confirm this hypothesis in the horse (49).

The absolute values of mean angular velocity for the three joints of interest were significantly greater during the retraction phase than during the protraction phase of the swimming cycle. Water resistance experienced by the forelimbs during protraction may contribute to such a reduction in the angular velocities during the protraction phase. Furthermore, during overground exercise, the forward movement (swing) of the distal forelimb is passive because of the release of elastic energy stored in tendons (50). As no stance phase occurs during swimming (non-load-bearing exercise), this observation raises questions about the kinetics and energy storing/releasing behavior of the limbs during swimming retraction and protraction. The protraction during swimming, corresponding to the swing phase of the ground stride, may be less efficient in energy preservation and may lead to an earlier fatigue.

### Limitations and Opportunities for Future Studies

Kinematic analysis of limbs underwater is technically challenging and there were limitations to the methodologies utilized. For example, the use of skin markers is prone to some degree of error as the skin moves over the joints. However, similar markers have been used successfully to track the movement of joints and measure angular parameters in horses during water treadmill exercise (12, 13). Furthermore, underwater images may be distorted due to light reflection and refraction. Finally, proper lighting underwater represents a challenge for studies of this kind. We strongly recommend that any new equine pool projects should take into account in the design a strong underwater lighting if further equine kinematics analyses are desired. Also, for future studies, other technology including inertial motor sensor to get 3D information without any visual data analysis could be preferred (14).

One objective of the present study was to compare swimming kinematics to a reference, the maximal passive ROM. However, ground gaits are often used during rehabilitation of various injuries, including joints. Comparing swimming kinematics to walk, trot and canter would also have been very relevant and useful for designing rehabilitation protocols. Such comparisons should be encouraged in future swimming kinematics studies.

Kinematic analysis of the limbs is important to establish a reference before further investigations and evidence-based rehabilitation protocols are designed. However, it has previously been hypothesized that the role of the forelimb is to assist with balancing the horse and determining the direction the horse swims in, while the hindlimb provides propulsion (51, 52). Interestingly, one horse was removed from the analysis because it showed almost no swimming movement of the forelimbs, which may corroborate with the non-propulsive role of the forelimbs during locomotion. This mare was only using her forelimbs every 15–20 cycles of motion where her body balance or her direction appeared affected.

During overground exercise, increased joint flexion may be obtained through specific exercises, such as trotting over poles (53). If the peak vertical forces observed during trotting over poles are not greater than those observed during regular trot (54), swimming enables horses to achieve increased flexion without any loading. While limited data on swimming equine is available, empirical use of swimming has provided encouraging results on that aspect (6). Even though the ability of swimming to improve fitness or performance was not assessed, the findings presented in the present study indicate the potential of swimming for rehabilitation of specific orthopedic injuries. Protraction of the forelimb and elevation of the forequarters is valued in some disciplines, such as dressage, especially in extended gaits. However, practicing such a gait results in greater fore fetlock extension during the retraction phase for instance (55) and therefore intensive practice of such an exercise may present a risk of accumulating stress and injury to the suspensory apparatus (56). Therefore, it is possible that the frequent use of swimming exercise, may help to develop or promote the elevation of the entire forelimb, while reducing the load applied to the joints and suspensory apparatus (57).

Controlled and blinded studies comparing the use of swimming for the rehabilitation of specific orthopedic injuries are warranted in the horse. Swimming could become an attractive rehabilitation tool at various stages post-injury that may allow faster and better healing of a wide range of orthopedic conditions, including fractures, joint and soft tissue injuries.

## Data Availability Statement

The raw data supporting the conclusions of this article will be made available by the authors, without undue reservation.

## Ethics Statement

The animal study was reviewed and approved by the Institutional Animal Care and Use Committee of the Equine Veterinary Medical Center, a member of Qatar Foundation, Doha, Qatar, under the protocol number EVMC-2020-1135. Written informed consent was obtained from the owners for the participation of their animals in this study.

## Author Contributions

ES: hypothesis generation and experimental design, interpreting and analyzing the results, writing, and revising the manuscript. RL, CR, and FD: hypothesis generation and experimental design, organizing and conducting the experiments, interpreting and analyzing the results, writing, and revising the manuscript. TV: experimental design, organizing and conducting the experiments, writing, and revising the manuscript. SF: experimental design, organizing and conducting the experiments, and revising the manuscript. SM and PM: organizing and conducting the experiments, writing, and revising the manuscript. SJ: organizing and conducting the experiments and revising the manuscript. All authors contributed to the article and approved the submitted version.

## Funding

This work was supported by the Equine Veterinary Medical Center, Member of Qatar Foundation - Intramural grant program; grant number RG19_FD1 (FD) and Faculty of Veterinary Medicine, University of Calgary, Chair in Equine Sports Medicine (RL).

## Conflict of Interest

The authors declare that the research was conducted in the absence of any commercial or financial relationships that could be construed as a potential conflict of interest.

## Publisher's Note

All claims expressed in this article are solely those of the authors and do not necessarily represent those of their affiliated organizations, or those of the publisher, the editors and the reviewers. Any product that may be evaluated in this article, or claim that may be made by its manufacturer, is not guaranteed or endorsed by the publisher.

## Footnotes

^1^GoPro Hero7 Silver, GoPro Inc., USA.

^2^Sudocrem, Forest Tosara LTD., Ireland.

^3^Canon EOS M50, Canon Inc., Japan.

^4^KINOVEA version 0.9.1., Available online at: http://www.kinovea.org

^5^R version 3.5.2.

## Supplementary Material

Supplementary Figure 1 **(A)** Atypical swimming style - This horse relied solely on its hind quarters to swim and kept both front limbs in a semi-flexed position most of the time with only a forelimb swimming movement every 15–20 hind limbs cycles. This horse was excluded from the analysis for obvious reasons. **(B)** Atypical swimming style - This horse kept its forelimb fully extended during part of the protraction phase. This was similarly observed for both the left and right forelimbs. This horse was included in the analysis.
