# Supplementary figures and images for: Kinematic Analysis During Straight Line Free Swimming in Horses: Part 1 - Forelimbs
Source: Front Vet Sci. 2021 Oct 14;8:752375. doi: 10.3389/fvets.2021.752375 (PMC8553013; doi:10.3389/fvets.2021.752375)

A

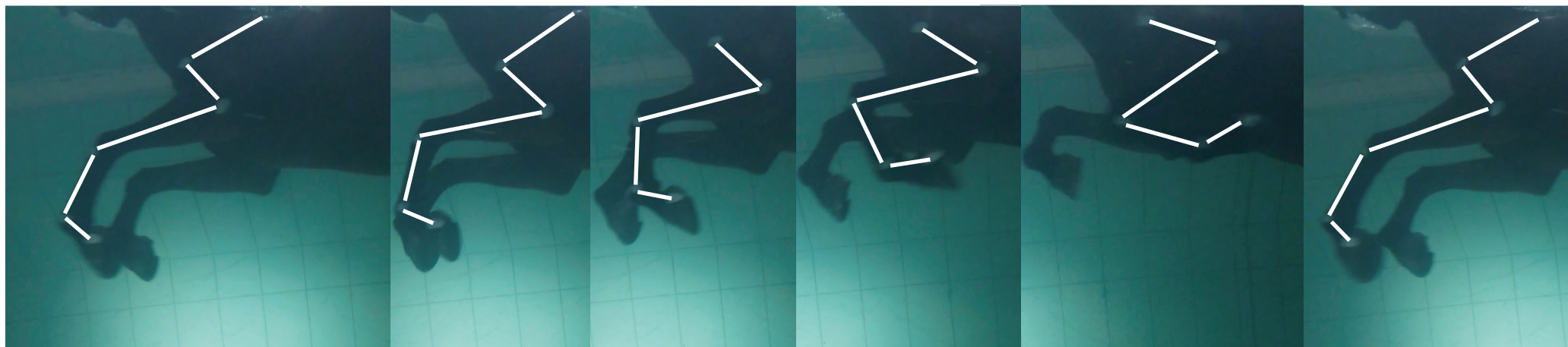

B

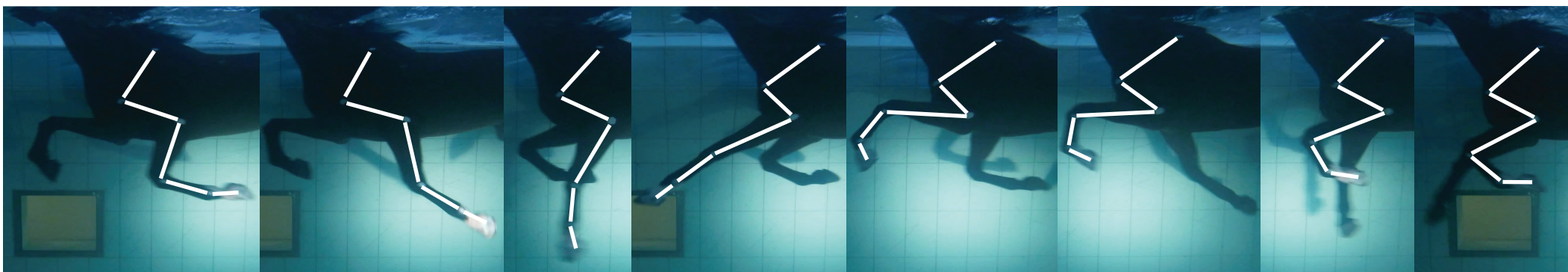

Supplement: Supplementary Figure 1 — (A) Atypical swimming style - This horse relied solely on its hind quarters to swim and kept both front limbs in a semi-flexed position most of the time with only a forelimb swimming movement every 15–20 hind limbs cycles. This horse was excluded from the analysis for obvious reasons. (B) Atypical swimming style - This horse kept its forelimb fully extended during part of the protraction phase. This was similarly observed for both the left and right forelimbs. This horse was included in the analysis. [file Data_Sheet_1.PDF]
